# Modeling physiological responses induced by an emotion recognition task using latent class mixed models

**DOI:** 10.1371/journal.pone.0207123

**Published:** 2018-11-16

**Authors:** Federica Cugnata, Riccardo Maria Martoni, Manuela Ferrario, Clelia Di Serio, Chiara Brombin

**Affiliations:** 1 University Centre of Statistics in the Biomedical Sciences (CUSSB), Vita-Salute San Raffaele University, Milan, Italy; 2 Department of Clinical Neurosciences, IRCCS San Raffaele Turro, Milan, Italy; 3 Department of Electronics, Information and Bioengineering (DEIB), Politecnico of Milan, Italy; University of Lleida, SPAIN

## Abstract

Correctly recognizing emotions is an essential skill to manage interpersonal relationships in everyday life. Facial expression represents the most powerful mean to convey important information on emotional and cognitive states during interactions with others. In this paper, we analyze physiological responses triggered by an emotion recognition test, which requires the processing of facial cues. In particular, we evaluate the modulation of several Heart Rate Variability indices, collected during the Reading the Mind in the Eyes Test, accounting for test difficulty (derived from a Rasch analysis), test performances, demographic and psychological characteristics of the participants. The main idea is that emotion recognition is associated with the Autonomic Nervous System and, as a consequence, with the Heart Rate Variability. The principal goal of our study was to explore the complexity of the collected measures and their possible interactions by applying a class of flexible models, i.e., the latent class mixed models. Actually, this modelling strategy allows for the identification of clusters of subjects characterized by similar longitudinal trajectories. Both univariate and multivariate latent class mixed models were used. In fact, while the interpretation of the Heart Rate Variability indices is very difficult when considered individually, a joint evaluation provides a better description of the Autonomic Nervous System state.

## Introduction

The ability to correctly recognize own and others’ emotions has been acknowledged as crucial for successful interaction with others. To assess the ability in understanding others’ mental states, psychometric tools, affective picture database and facial expression database have been developed (see for an overview, [1]) and used in combination with physiological monitoring [2].

Cognitive stress triggered by emotion recognition task affects the Autonomic Nervous System (ANS) and, as a consequence, Heart Rate Variability (HRV).

In this perspective, one can observe that HRV is a noninvasive marker reflecting the ANS activity on the heart.

Starting from Porges’ polyvagal theory, which links ANS activity and successful social engagement behaviors [3, 4], Quintana *et al*. (2012) [2] suggested the existence of a relationship between the resting-state HRV and the performance on the Reading the Mind in the Eyes Test (RMET, [5]), which is a basic emotion recognition task, widely used also in clinical context [6, 7].

The results obtained by Quintana *et al*. (2012) were further explored in clinical and non clinical samples by Shahrestani *et al*. (2014) [8]. The HRV is widely used in psychiatry as a transdiagnostic marker [9]: this explains the recent push to establish neurobiological markers of psychiatric illness for improving nosology [10]. Meta-analyses have established that individuals with a range of psychiatric disorders have a reduced HRV [11–13]. Several studies have evaluated the changes in the ANS during the RMET task in clinical samples [14–16]

The novelty of this work is to evaluate the impact of an emotion recognition task on physiological response, during its completion, considering the effect and the difficulty of each stimulus, while accounting for standard confounding clinical and psychological variables as in Quintana et al. (2012).

In particular, we expect that a more difficult task in emotion recognition will activate a stress response, i.e. an activation of the sympathetic nervous system branch of the ANS. A typical sign of such activation is the increase of HR values.

Since the focus is on physiological modifications over time, statistical models for longitudinal data are needed. In particular, changes from baseline in HRV during the RMET test were modeled by means of Latent Class Mixed Models (LCMMs, [17]). This modeling strategy allows to manage non Gaussian continuous and ordinal outcomes. Differently from standard Linear Mixed Effects models (LME), LCMMs account for heterogeneous profiles of the longitudinal outcome, thus uncovering homogeneous subpopulations within a larger heterogeneous population.

In this work we applied flexible models to address two goals. First, we evaluate whether successful emotion recognition elicits a physiological activation, while accounting for demographic/clinical characteristics and psychopathological traits. Second, we identify clusters of subjects characterized by similar longitudinal trajectories.

Moreover, within the same framework, we jointly model biosignals, as multivariate outcomes potentially underlying a common latent trait described as an “overall physiological activation”. From a physiological perspective modeling several biosignals jointly, instead of separately, provides an integrated view of the autonomic physiological response pathway.

The paper is organized as follows. In the first part, sample description is provided along with the illustration of the experimental sessions and the description of the collected psychometric and physiological measures. Then, in the Methods Section, Rasch model and Latent Class Mixed Models are described. Selected results and concluding remarks are finally discussed.

## Sample and experimental session description

The Reading the Mind in the Eyes Test (RMET) is an advanced test used to measure Theory of Mind (ToM) abilities. In particular, it is suited to index emotion recognition aptitude. It consists of 36 black and white images of the eye region of different faces, and participants are asked to choose among four possible mental states to describe the person whose eyes are pictured. It easily allows to evaluate the ability of accurately identifying others’ mental states.

334 subjects taken from the general population completed a computerized version of the original “pencil and paper” RMET test. Out of these, 174 were females and 160 were males, with an average age of 30.2 years (sd = 10.34, range = 18-68ys). 91 subjects out of the 334 subjects completed the online version of the RMET in a laboratory (44 females and 47 males, average age 26.78 years, ranging from 18 to 52ys). During the experimental session, physiological reactivity has been monitored at rest and while completing RMET. Various indices of HRV, skin conductance and blood volume pulse have been collected and derived. In this work, we focus on HRV modulation induced by the emotion recognition task. In particular, biosignals in the time-domain, e.g. mean and standard deviation of beat to beat (R-R) intervals and various spectral indices of HRV, were extracted.

Experimental sessions were organized in the morning at 11 a.m. (±1 h) and in the afternoon at 3 p.m (±1 h). Electrodes were placed in the non-dominant hand and forearms and physiological baseline was recorded for 5 minutes (at rest). Biosignals were measured, amplified, and recorded using Procomp InfinityTM (Thougth Technology, USA). After baseline measurement, RMET was administered: RMET items, i.e., 1 trial + 36 target pictures, were shown on a monitor according to the original sequence. Before showing each new stimulus, a slide with a fixation point was displayed for 3 seconds. Immediately after the presentation of the stimulus and the selection of the emotional state conveyed by the eyes, a black slide appeared and remained on the screen for 5 seconds before the fixation point slide.

Several self-report questionnaires for the assessment of anxiety, depression severity, alexithymia and the presence of psychopathological traits related to obsessive-compulsive or eating disorders have been also administered once completing the test. These factors have been showed to have an impact on physiological activation [18–22].

In particular, the State-Trait Anxiety Inventory (STAY-Y, [23]) has been administered to measure trait (STAI-1) and state anxiety (STAI-2). Anxiety Sensitivity (AS) construct was assessed by means of the Anxiety Sensitivity Index (ASI, [24]). Depression severity was evaluated by means of the Beck Depression Inventory (BDI-II, [25]). Psychopathological traits related to obsessive-compulsive or eating disorders were assessed using respectively the Padua Inventory (PI, [26]) and the Eating Disoder Inventory-2 (EDI-2, [27]). Actually, PI was designed to measure four factors, namely “Becoming Contaminated”, “Checking Behaviours”, “Impaired Control of Mental Activities”, “Urges and Worries of Losing Control”. Moreover, alexithymia, i.e., the difficulty in identifying and describing emotions, was measured by means of the Toronto Alexitimia Scale (TAS-20, [28]). The questionnaire has a three-factor structure. The first factor (F1) assesses the ability to identify feelings and to distinguish them from the somatic sensations that accompany emotional arousal. The second factor (F2) assesses the ability to describe feelings to other people, while the third (F3) evaluates externally oriented thinking.

### Ethical statement

All the procedures performed in this study involving human subjects were conducted in accordance with the ethical standards of the San Raffaele Hospital and with the 1964 Helsinki declaration and its later amendments or comparable ethical standards. With reference to the online administration of the RMET, the questionnaire was completed anonymously without collecting any sensitive data compromising identities of the respondents. The participation to the experimental sessions was voluntary and, prior to study participation, participants gave their written informed consent. The entire FIRB project, of which the study presented in the paper is a part, was approved by the Ethics Committee of San Raffaele Hospital (CE 1129 register 213/2014).

### HRV analysis

HRV refers to the variability of the length of beat to beat (R-R) intervals in electrocardiograms. It can be quantified by descriptive statistics of R-R interval duration and its variation over time, i.e. range, mean and standard deviation. Beat-by-beat series of R-R intervals were obtained by ECG recordings by applying the freely available “eplimited” software [29]. Each recording was subdivided into several segments: the baseline epoch, i.e. the 5 minutes before the starting of the questionnaire, and the segments of variable length associated to the period of time following the vision of the stimulus. Only when 85% of beats resulted normal according to ECG quality and R-R physiological value range, the segments were analyzed. The spectral analysis was performed for the baseline recording only. For each segment and the baseline epoch we computed the following time-domain parameters [30]: mean of beat to beat (R-R) intervals (msec), the average heart rate in beat per minute (bpm), the standard deviation of beat to beat (R-R) intervals commonly named SDNN, the square root of the sum of the squares of differences between adjacent R-R intervals (RMSSD), the number of pairs of adjacent R-R intervals differing by more than 50 ms in the sequence (NN50), the sample asymmetry represented by the ratio R1/R2 [31], the SD1 and SD2 parameters from the Poincaré plot [32]. Poincaré plot is actually a diagram in which each R-R interval of tachogram is plotted against the previous R-R interval, where the values of each pair of successive R-R interval define a point in the plot.

All the collected time domain measures of HRV are summarized in Table 1.

**Table 1:** Heart rate variability measures.

Time-domain	
Measure	Description
meanRR(msec)	Mean of beat to beat (R-R) intervals
std(msec)	Standard deviations of beat to beat (R-R) intervals
RMSSD	Square root of the mean of the squares of differences between adjacent beat-to-beat intervals
SDSD	Standard deviation of the successive differences of the R-R intervals
NN50	Number of pairs of successive normal-to-normal (NN) intervals that differ by more than 50 ms.
pNN50	Percentage of differences between adjacent NN intervals that are greater than 50 ms
mean(bpm)	Average heart rate in beat per minute
R1/R2	Sample asymmetry, given by the ratio of two measures, each the weighted sum of values less than (R1) or greater than (R2) the median R-R interval.
SD1	Dispersion of points perpendicular to the axis of line of identity in the Poincaré plot
SD2	Dispersion of points along the axis of line of identity in the Poincaré plot

## Statistical methods

### Identifying difficult RMET questions

Differently from previous works which evaluate the association between HRV and emotion recognition indexed by RMET total score (e.g., [2]), we decided to use the information provided by each item of the test and, in particular, to examine the effect of item difficulty on HRV modulation. Hence, Rasch model was used, on the total sample, just to estimate item difficulty, thus allowing for a classification of items as “difficult” or “easy”. The model has been proposed in the psychometric field to study the ability of a subject to overcome or fail a test. The key hypothesis underlying the Rasch model is that the probability of a correct answer depends on two parameters: a parameter for items and a parameter for the subject. For the sake of simplicity, a binary Rasch model without accounting for guessing was used. Incorrect answers were aggregated into a single category. The probability that an individual with a particular trait level will correctly answer an item characterized by a particular difficulty is

$$\begin{array}{c} \mathrm{Pr}\left(X_{ij}=1\right)=\frac{\text{exp}(\theta_{i}-\beta_{j})}{1+\text{exp}(\theta_{i}-\beta_{j})},\;i=1,\ldots ,N\;\text{subjects},j=1,\ldots ,J\;\text{items} \end{array}$$

where *β*_*j*_ are the *item parameters* measuring the difficulty of the item and *θ*_*i*_ represent the *person parameter* measuring the ability of the respondent [33]. Estimated difficulty parameters were used to classify items into two categories, respectively as “difficult” or “easy”, if their values were larger or smaller than zero, which is the mean of the latent trait.

### Latent class mixed models

LCMMs provide a flexible framework to model Gaussian or non-Gaussian (curvilinear) quantitative and even ordinal longitudinal outcomes.

LCMMs generalize traditional LMEs, assuming that the population is heterogeneous and *G* unobserved sub-populations (latent classes), with their own mean profiles of trajectories, may be identified. Hence these models allow to account for common fixed effects over classes, for class-specific fixed effects and for sources of unobserved heterogeneity by specifying random effects. Following the notation provided by Proust-Lima *et al*. (2015) [17, 34] and consistently with the literature on latent variable modelling, the approach requires the specification of a structural latent model, i.e., a standard linear mixed model without measurement errors, along with a measurement model, linking the latent process to the outcome of interest. When heterogeneous population is assumed, for a subject *i* belonging to the class *c*_*i*_ equal to *g* (*g* = 1, …, *G*), a latent class-specific process can be defined as

$$\begin{array}{c} \Lambda_{i}{\left(t\right)|}_{c_{i}=g}=X_{1i}{\left(t_{ij}\right)}^{\prime }\beta +X_{2i}{\left(t_{ij}\right)}^{\prime }\gamma_{g}+Z_{i}{\left(t_{ij}\right)}^{\prime }u_{ig}+w_{i}\left(t_{ij}\right) \end{array}$$

where

- *t*_*ij*_ denotes the time of measurement for subject *i* (*i* = 1, …, *N*) at occasion *j* (*j* = 1, …, *n*_*i*_)
- *X*_1*i*_(*t*_*ij*_) and *X*_2*i*_(*t*_*ij*_) are vectors of time-dependent covariates respectively with common fixed effects *β* over classes and class-specific fixed effects *γ*_*g*_
- *Z*_*i*_(*t*_*ij*_) is a vector of time-dependent covariates associated with individual class-specific random effects *u*_*ig*_
- *w*_*i*_(*t*_*ij*_) represents an autocorrelated process.

Then a measurement model ruling the relationship between the longitudinal outcome variable, observed at time *t*_*ij*_, and the latent process is defined as it follows

$$\begin{array}{c} Y_{ij}{|}_{c_{i}=g}=H\left(\Lambda_{i}\left(t\right){|}_{c_{i}=g}+\epsilon_{ij};\eta \right) \end{array}$$

where *H* is a parametrized monotonic increasing link function, defining linear/nonlinear transformations, *ϵ*_*ij*_ are independent normally distributed errors and represents a noisy latent process at time. Every subject is assigned to one latent class only. For each subject, the latent class membership is described by a latent variable *c*_*i*_ that equals *g* if *i* belongs to class *g* and probability of latent class membership is modeled using a multinomial logistic regression:

$$\begin{array}{c} \pi_{ig}=P\left(c_{i}=g|X_{3i}\right)=\frac{e^{\xi_{0g}+X_{3i}^{\prime }\xi_{1g}}}{\sum_{l=1}^{G}e^{\xi_{0l}+X_{3i}^{\prime }\xi_{1l}}} \end{array}$$

where *ξ*_0*g*_ is the intercept for class *g* and *ξ*_1*g*_ is the vector of class-specific parameters related to the time-independent covariates *X*_3*i*_.

### LCMM for multivariate outcomes case

LCMM framework has been generalized to the case of multiple outcomes measuring the same latent process [34]. Let us assume that *K* longitudinal outcomes, indicators of the same underlying construct, are available. For each subject *i*, *i* = 1, …, *N*, and outcome variable *k*, *k* = 1, …, *K*, the set of measurements *y*_*ik*_ = (*y*_*i*1*k*_, …, *y*_*ijk*_, …, *y*_*in*_*ik*_*k*_)′ is collected at times *t*_*i*1*k*_, …, *t*_*ijk*_, …, *t*_*in*_*ik*_*k*_.

This model specification allows for a number of measurements and related time records varying within subjects and outcomes.

Observed outcomes actually should provide information on the *true* common latent process $\Lambda_{i}{\left(t\right)}_{t\in R}$.

As for the univariate case, a *measurement model* for each collected outcome and a *structural model* for the latent process can be defined.

The first describes the association between the observed outcome and the underlying latent process, the second allows to examine changes in the latent trait over time, thus identifying variables modulating the construct of interest.

The measurement model can be specified as it follows

$$\begin{array}{c} H_{k}\left(y_{ijk};\eta_{k}\right)={\tilde{y}}_{ijk}=\Lambda_{i}\left(t_{ijk}\right)+X_{2i}{\left(t_{ijk}\right)}^{\prime }\gamma_{k}+\alpha_{ik}+\epsilon_{ijk} \end{array}$$ (1)

where *H*_*k*_(⋅; ***η***_*k*_) is a flexible *outcome-specific* parameterized *link function* transforming *y*_*ijk*_ into an intermediate Gaussian variable ${\tilde{y}}_{ijk}$; Λ_*i*_(*t*_*ijk*_) is the true *common* latent process at time *t*_*ijk*_; **X**_2*i*_(*t*_*ijk*_)′ and *γ*_*k*_ are, respectively, *time-dependent covariates* and *contrasts* accounting for differential effects of covariate/time on outcomes after adjustment for the latent process level. Finally, *α*_*ik*_ are subject- and test-specific *random effects* and *ϵ*_*ijk*_ are *measurements errors*.

Several different link functions (linear, splines, thresholds, etc.) can be chosen depending on the type of the longitudinal markers. Curvilinear as well as bounded quantitative longitudinal outcomes can be analyzed within this modeling framework.

The *structural model*

$$\begin{array}{c} \Lambda_{i}\left(t\right)=X_{1i}{\left(t\right)}^{\prime }\beta +Z_{i}{\left(t\right)}^{\prime }b_{i}+w_{i}\left(t\right) \end{array}$$

accounts for the dynamic nature of the latent trait, embodying information on collected covariates and time. Actually, **X**_1*i*_(*t*_*ijk*_)′ are *time-dependent covariates* associated with fixed effects *β*, **Z**_*i*_(**t**)′ are other *time-dependent covariates* associated with random effects **b**_*i*_. Autocorrelated process ***w***_*i*_(*t*) may be also defined. As in the univariate LCMM, also in the multivariate framework, latent classes of subjects may be hypothesized.

**Fig 1:**
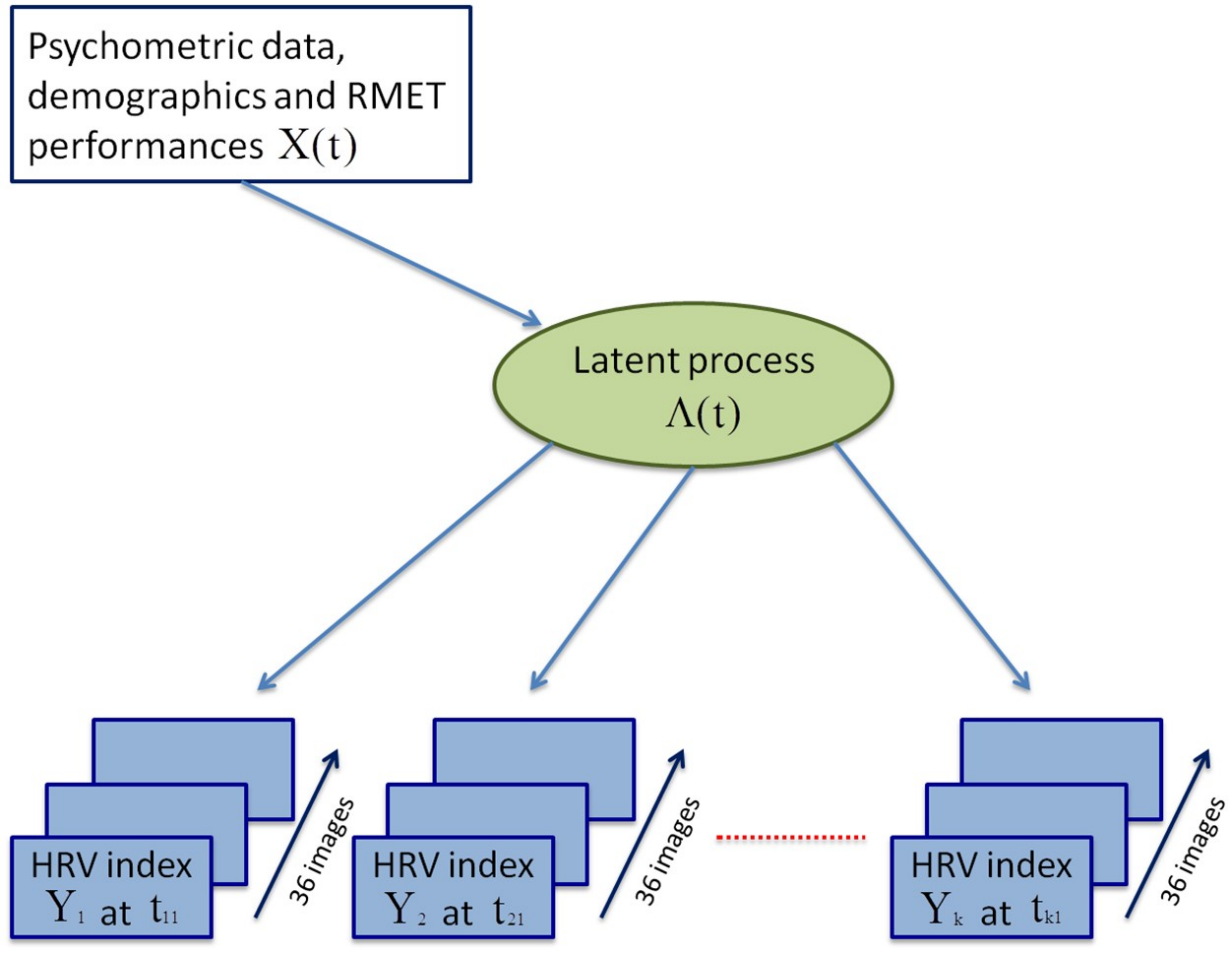
Example of structural and measurement models in a multivariate modeling framework, where several outcomes are expected to measure the same phenomenon. In a very general experimental setting where elicited physiological reactions are measured, one may assume to have different HRV indices measured in several occasions, e.g., while administering emotionally charged stimuli. These multivariate outcomes potentially underlie a common latent trait that could be described as an “overall physiological activation”, which in turn is affected and modulated by demographic and clinical characteristics.

A sketch showing the idea underlying this modeling procedure is provided in Fig 1, where multivariate outcomes are represented by different HRV indices.

## Model specification

Among all the HRV indices, we chose a measure of central tendency and one of variability of beat to beat (R-R) intervals since other measures resulted correlated with each others and therefore redundant. To account for possible individual-specific physiology, the baseline values of the collected indices were subtracted to the actual values recorded while administering the RMET, thus allowing to highlight the activation induced by the task itself (if any exists).

We first fitted separate LCMM models modelling to separately examine the changes, with respect to baseline values, of the average heart rate expressed as beat per minute (Δ*mean.bpm*) and of the standard deviations of R-R intervals (Δ*std.msec*). Then, we model them jointly. In all models, we estimated the impact of the following variables on the physiological response:

- item (i.e., picture) sequence as *time* variable
- performance on the RMET test (correct/wrong answer) and item difficulty (as categorical variable)
- demographics and clinical characteristics (age, gender, state anxiety, depression, alexithymia)

Moreover, we included interaction terms to evaluate gender-specific effects of clinical variables on physiology. In particular, we tested for differential effects of anxiety, depression and alexithymia depending on respondent’s gender. All the above mentioned covariates were set as fixed in the model. Random intercept and random slope models were specified. RMET pictures sequence was set as random effect in the latent process mixed model. Random effects were grouped by subject’s ID.

Flexible splines link functions were considered to account for nonlinearities in the longitudinal response.

LCMMs were estimated with a number of latent classes ranging from 1 to 4 in order to ensure an adequate sample size in each class and thus allowing for accurate parameter estimates [35]. Bayesian information criterion (BIC, [36]) was used to choose the optimal number of latent classes, thus following traditional mixture modeling approaches [37].

Whenever the best model included at least two latent classes, we test, by applying appropriate inferential procedures, whether subjects assigned to different latent classes were different respect to clinical or demographic characteristics. We used this strategy to reduce the number of variables to be included in the class membership model. We aim so at reducing computational burden and improving model convergence. Covariates effectively distinguishing among latent classes were included to estimate the model and BIC was used for model comparison. Hence, we compared simpler intercept-only model with more complex models including also covariates in the class-membership multinomial logistic model.

All the analyses were performed using R Statistical Software [38], version 3.3.1. In particular *lcmm* [34] package was used to estimate latent class mixed models.

## Results

When examining the simplest LCMM model with 1 latent class, we found that item difficulty, BDI and TAS play a significant role in modulating the change with respect to baseline of average beats per minute (Δ*mean.bpm*) during the emotion recognition task. In particular, in presence of difficult items, physiological response significantly increases (see Table 2).

**Table 2:** Separate simple LCMM models, with 1 latent class, for the index Δ*mean.bpm* and Δ*std.msec*. BDI.TOT indicates the total score in the Beck Depression Inventory, i.e. the questionnaire administered to evaluate depression severity, STAI.Y.1 indicates the state anxiety measured by the State-Trait Anxiety Inventory, TAS.TOT is the total score in the Toronto Alexitimia Scale used to measure alexithymia, i.e., the difficulty in identifying and describing emotions. “Easy” category was chosen as reference in the item difficulty variable derived from Rasch model and “wrong” as reference for the item answer.

Parameter	Average Beats Per Minute			Sd of RR intervals		
	Estimate	SE	*p*-value	Estimate	SE	*p*-value
Intercept (not estimated)	0			0		
Item difficulty	0.1148	0.0381	**0.0026**	0.0912	0.0385	**0.0179**
Time	0.0045	0.0040	0.2628	0.0079	0.0028	**0.0044**
Correct answer	0.0653	0.0418	0.1183	-0.0134	0.0422	0.7508
Age	-0.0163	0.0129	0.2068	-0.0056	0.0129	0.6656
Female:STAI.Y.1	-0.0062	0.0175	0.7233	0.0046	0.0176	0.7952
Male:STAI.Y.1	-0.0315	0.0164	0.0548	0.0173	0.0167	0.2998
Female:BDI.TOT	0.0153	0.0263	0.5602	0.0052	0.0268	0.8475
Male:BDI.TOT	-0.0816	0.0319	**0.0106**	-0.0362	0.0352	0.3027
Female:TAS.TOT	-0.0011	0.0140	0.9387	0.0102	0.0147	0.4871
Male:TAS.TOT	0.0322	0.0135	**0.0165**	-0.0001	0.0135	0.9918
BIC	15116.42			25235.75		

Moreover, in males, as TAS increases, Δ*mean.bpm* significantly increases, while increasing depression levels measured through BDI significantly decreases the variation in the physiological response.

To address the second goal of our research, that is the identification of clusters of homogeneous subjects, we estimated models with a number of latent classes greater than 1. When increasing the number of latent classes, we found that the two latent classes model is the best in terms of BIC, with 51 subjects assigned to class 1 and 35 to class 2 (BIC = 15114.07). These latent classes significantly differ on total levels of alexithymia (average class 1 TAS was 43.10± 9.08 while in class 2 it was 47.8± 10.45, Mann-Whitney test *p*-value = 0.038) and in terms of the third TAS subscale measuring “Externally-Oriented Thinking” (average class 1 TAS-F3 was 17.69± 4.32 while in class 2 it was 19.54± 4.71, Mann-Whitney test *p*-value = 0.031). When including these covariates in the class membership model, we found that the best model is the one with two latent classes and total alexithymia score as covariate in the class membership model (BIC = 15113.02). Results are shown in Table 3.

**Table 3:** LCMM for the index Δ*mean.bpm* with 2 latent classes (model BIC: 15113.028) and total alexithymia score as covariate in the class membership model.

Parameter	Estimate	se	*p*-value
**Class membership probability**			
Intercept class1	2.9679	1.2393	**0.0166**
TAS.TOT class1	-0.0582	0.0265	**0.0281**
**Longitudinal model**			
Intercept class1 (not estimated)	0.0000		
Intercept class2	-1.5786	0.1162	**0.0000**
Item difficulty	0.1153	0.0381	**0.0025**
time	0.0045	0.0040	0.2626
Correct answer	0.0661	0.0418	0.1134
Age	-0.0065	0.0083	0.4362
female:STAI.Y.1	-0.0075	0.0106	0.4765
male:STAI.Y.1	-0.0311	0.0097	**0.0014**
female:BDI.TOT	0.0067	0.0173	0.6981
male:BDI.TOT	-0.1092	0.0202	**0.0000**
female:TAS.TOT	0.0227	0.0088	**0.0096**
male:TAS.TOT	0.0547	0.0091	**0.0000**

TAS presents a significant effect on class membership, with more alexithymic subjects less likely belonging to class 1. Actually, subjects assigned to class 1 show, on average, a higher variation in the physiological response, with respect to the baseline, in the longitudinal process. In the longitudinal model, we found that item difficulty and increasing TAS levels significantly increases Δ*mean.bpm*. On the other side, with increasing levels of depression and anxiety, in men, the change in the physiological response decreases significantly.

Average posterior probabilities of falling into the class in which the subjects were classified are equal to 0.9464 and 0.9739 (Table 4), thus suggesting an unambiguous classification. In addition, the non-diagonal terms indicate that subjects classified in class 1 have a non-negligible probability of belonging to class 2 (mean of 0.0536) and conversely (mean of 0.0261).

**Table 4:** Mean of the posterior probabilities of belonging to each latent class in the model for Δ*mean.bpm*.

Final classification	Number of subject	Mean of the probabilities of belonging to each latent class	
		1	2
class 1	52	0.9464	0.0536
class 2	34	0.0261	0.9739

In the model with 1 latent class, evaluating changes, with respect to baseline, in the standard deviations of R-R intervals (Δ*std.msec*), we found that the presentation of items classified as difficult and the rating task itself positively affect the outcome: in presence of difficult pictures to be rated and, as the number of presented pictures increases, also the standard deviations of R-R intervals increases. Clinical covariates do not significantly modulate this signal (see Table 2). When increasing the number of latent classes, we found that a three latent classes model is the best choice in terms of BIC, with 32 subjects assigned to class 1, 40 to class 2 and 12 to class 3 (BIC = 25214.13).

Examining differences among these classes, it emerged that they significantly differ on the PADUA subscale reflecting “contamination fear” (average PADUA F2 score in class 1 was 9.22± 5.77, in class 2 was 7.15± 5.62 and in class 3 was 4.83±4.06, Kruskal-Wallis test *p*-value = 0.035, with class 3 resulting significantly different from class 1 in the post-hoc pairwise comparison). However, when including this covariate in the class membership model, we found that the best model is the only-intercept three latent class model, being the latter model associated with higher BIC.

We found that Δ*std.msec* significantly increases as difficult items were shown and as the number of presented and rated items increased (significant time effect). Clinical characteristics do not play a significantly role in the physiological response modulation (Table 5).

**Table 5:** LCMM for the index Δ*std.msec* with 3 latent classes (model BIC: 25214.13) and only-intercept class membership model.

Parameter	Estimate	se	*p*-value
**Class membership probability**			
Intercept class1	1.0157	0.3603	**0.0048**
Intercept class2	1.2139	0.3511	**0.0006**
**Longitudinal model**			
Intercept class1 (not estimated)	0.0000		
Intercept class2	0.8748	0.0850	**0.0000**
Intercept class3	-1.6399	0.1247	**0.0000**
Item difficulty	0.0915	0.0385	**0.0175**
Time	0.0079	0.0028	**0.0045**
Correct answer	-0.0146	0.0422	0.7300
Age	-0.0048	0.0059	0.4174
female:STAI.Y.1	0.0043	0.0075	0.5653
male:STAI.Y.1	0.0065	0.0076	0.3861
female:BDI.TOT	-0.0139	0.0132	0.2916
male:BDI.TOT	-0.0182	0.0175	0.2988
female:TAS.TOT	0.0099	0.0072	0.1713
male:TAS.TOT	0.0054	0.0061	0.3769

Average posterior probabilities are equal to 0.9236, 0.9308 and 0.9816, thus suggesting again an unambiguous classification (see Table 6).

**Table 6:** Mean of the posterior probabilities of belonging to each latent class in the model for Δ*std.msec*.

Final classification	Number of subject	Mean of the probabilities of belonging to each latent class		
		1	2	3
class 1	32	0.9236	0.0763	0.0002
class 2	40	0.0692	0.9308	0.0000
class 3	12	0.0184	0.0000	0.9816

When jointly modeling the two physiological indices (Δ*mean.bpm* and Δ*std.msec*), we found that a three latent classes model is the best in terms of BIC, with 32 subjects assigned to class 1, 41 to class 2 and 13 to class 3 (BIC = 41980.85).

If we assume that the latent trait measured by these two indices is a “global activation”, we may conclude that the length of the task (the increasing number of presented pictures) and the presentation of difficult items have a positive and significant impact on the latent trait (see Table 7). Inspecting the posterior probabilities table, we can observe an unambiguous classification (see Table 8).

**Table 7:** Jointly modeling of Δ*mean.bpm* and Δ*std.msec* (3 latent classes, BIC = 41980.85).

Parameter	Estimate	se	*p*-value
**Class membership probability**			
Intercept class1	0.9125	0.3713	**0.0140**
Intercept class2	1.1823	0.3560	**0.0009**
**Longitudinal model**			
Intercept class1 (not estimated)	0.0000		
Intercept class2	1.6268	0.2699	**0.0000**
Intercept class3	-3.0444	0.4841	**0.0000**
Item difficulty	0.1776	0.0752	**0.0182**
Time	0.0150	0.0055	**0.0065**
Correct answer	-0.0223	0.0775	0.7740
Age	-0.0084	0.0108	0.4410
femaleSTAI.Y.1	0.0067	0.0140	0.6317
maleSTAI.Y.1	0.0124	0.0160	0.4396
femaleBDI.TOT	-0.0283	0.0319	0.3743
maleBDI.TOT	-0.0561	0.0455	0.2174
femaleTAS.TOT	0.0169	0.0176	0.3366
maleTAS.TOT	0.0090	0.0121	0.4575

**Table 8:** Mean of the posterior probabilities of belonging to each latent class in the multivariate model.

Final classification	Number of subject	Mean of the probabilities of belonging to each latent class		
		1	2	3
class 1	32	0.8895	0.0960	0.0145
class 2	41	0.0674	0.9326	0.0000
class 3	13	0.0379	0.0180	0.944

## Discussion

In this work we investigate the role of an emotion recognition task on HRV, accounting also for demographic and clinical characteristics. LCMMs provide an appealing framework to analyse univariate and multivariate longitudinal psychophysiological outcomes, due to their flexibility in handling non-Gaussian continuous outcomes. Differently from standard models for longitudinal data, the proposed approach may account for a heterogeneous population. The HRV is a well-established marker of the ANS activity. However, the interpretation of single HRV indices may be complex. Alternatively, a set of indices may provide a better description of the ANS activation or state. The typical example is given by the rest tilt test, which is a provocative sympathetic stimulus (see Figure 5 in [30]) where the physiological response is described as an increase of the heart rate along with a decrease of the standard deviation. However, when the heart rate increases, without a joint reduction of standard deviation, the interpretation of an exclusive activation of the sympathetic nervous system (SNS) could not still hold.

Our modelling strategy highlighted that in presence of complex stimuli, reflecting complex mental states to recognize, physiological response significantly increases. In fact, in both the models for Δ*mean.bpm* (Average Beats Per Minute) and Δ*std.msec* (standard deviations of R-R intervals), as well as in the joint model, we found a significant and positive effect of item difficulty. This result confirms previous findings reported by Park *et al*. (2013) [39], which suggested how cardiac vagal tone is associated with more adaptive top-down and bottom-up modulation of emotional attention to faces. In this perspective, our results highlight how the recognition of more complex faces (e.g., the recognition of more complex emotional states) require more physiological activation. Future studies should investigate the performance and difficulties of subjects with psychopathologies once exposed to these complex stimuli.

Moreover, we found that alexithymic features are associated with an increase in Δ*mean.bpm*.

These results lead to some speculation from the psychological perspective.

Grynberg *et al*. (2012) [40] reviewed and supported the hypothesis that alexithymia is linked to deficits in processing and labeling emotional facial expressions. In particular, the Authors suggested that alexithymia would be associated with processing deficits already at the perceptual level. Our data could deepen this hypothesis since it supports the belief that a bottom-up activation is present during the evaluation of an emotion inducing facial expression. Hence, a deficient perceptual level would be associated with a bottom-up physiological activation.

Finally, in line with the current literature, we found that anxiety and mood state influenced the physiological response during the task in males. Neuroimaging studies have well documented a gender dimorphism for what concerns limbic system in humans. More in detail, amygdala is modulated by both vasopressin (involved in anxiety and stress) and oxytocin [41] and it is able to influence the autonomic control [42, 43]. Moreover, lots of evidences [44–46] supported a female superiority in the ability to read others mental states, linking this capability with the action of gender-related hormones. Taken together these evidences suggest that it is crucial to control for depression and anxiety when analyzing ANS arousal during an emotion recognition task as proposed by Quintana and colleagues in their previous work. Future studies should be conducted to deepen the knowledge on the complex interrelationships among sex hormones, anxiety, depression, the ability to understand other mental states and ANS arousal. The modeling approach here proposed can be applied to provide an insight into this mechanism.

Our work provides evidence for a relationship between emotion recognition and HRV during RMET, by evaluating longitudinal trajectories of physiological responses during the task. Our methodology should be considered a first step to provide clinicians the chance to investigate theory of mind performances and its physiological components. In fact, even if it is well documented the role of theory of mind as mediator of effectiveness in psychotherapy treatments [47] as well as it is known the role of ANS in the maintenance of psychopathological features [48], nowadays there is lack of methodologies able to assess their relationship and possible interactions.

To conclude, some directions for future works can be suggested. From the statistical point of view, we would rather consider a more complex Rasch model appropriate for multiple-choice test thus obtaining a more precise estimate of item difficulty. Moreover, the collection of more “stress-coping related” covariates could improve the characterization of the latent classes.

Finally, we focused mainly on HRV parameters. However, electrodermal activity could be also used in evaluating stress levels induced by the task. Actually, skin electrical conductivity depends on the activity of eccrine sweat glands which, in turn, is controlled by the nervous system and is involved in thermoregulation processes or varies in response to stressful situations. Also skin conductance response reflects the ANS activity and has been widely used as a marker of emotional states [49, 50].
